# It all clicks together: In silico drug discovery becoming mainstream

**DOI:** 10.1002/ctm2.766

**Published:** 2022-04-04

**Authors:** Antonina L. Nazarova, Vsevolod Katritch

**Affiliations:** ^1^ Department of Quantitative and Computational Biology Department of Chemistry Bridge Institute USC Michelson Center for Convergent Biosciences University of Southern California Los Angeles California USA

*In silico*, or *computer‐aided* drug design (CADD) has been around for a few decades, experiencing several waves of hype and disillusionment. The idea that computational modelling of chemical compounds binding to and modulating their receptor targets could someday replace tedious and expensive high‐throughput screening assays for hit discovery, as well as custom synthesis of hundreds of derivatives for lead optimization, has always been very attractive. Of course, assays and synthesis still would be needed, but in silico predictions would help to dramatically narrow down the number of compounds to make and assay in the test tube. Although there were quite a few success stories along the way, the general economics of computationally driven drug discovery was never made to work at scale in the past.

The last couple of years, however, show signs of a tectonic shift toward embracing in silico drug discovery in both academia and industry.^1^ Big Pharma and Biotech are expanding their CADD teams, smaller Biotech companies are hiring their first computational chemists, and many new startups include a major computational component as part of their business plans. Moreover, large and small startups are popping up like mushrooms, where business models heavily rely on computational technologies, which is often a combination of advanced molecular modelling with machine learning and artificial intelligence. Is it just a new wave of hype or the CADD technology has matured enough to become a mainstream part of the drug discovery process? There are several reasons to believe that several key components of CADD, especially based on molecular modelling technologies, have recently "clicked together" to make it scientifically and economically viable, even in competition with ever‐growing in vitro technologies like DNA‐labelled libraries.

These key components include (1) *greatly improved availability and accessibility of structural information for drug targets*. With more than 190,000 protein structures in PDB,^2^ structures are available for most clinically relevant targets or at least for their close orthologs. Especially dramatic in the last decade was the structural revolution for GPCRs targets, the large membrane receptor family that includes ∼30% of all clinically relevant targets.^3^, ^4^ If it is not in PDB yet, soluble proteins are now routinely solved by crystallography in a few months at the inception of a drug discovery project. The invention of high‐resolution cryo‐EM in the last few years has made solving large and membrane‐embedded proteins and complexes that are also readily accessible.^5^, ^6^ (2) *Chemical libraries have grown in diversity and scaled to tens of millions*, making their physical screening impractical. Moreover, the last few years brought about ultra‐large libraries, comprising hundreds of millions,^7^, ^8^ or even billions^9^ of compounds. These libraries are, of course, virtual because synthesizing and storing individual compounds at this scale is not feasible. These virtual compounds, however, are based on highly optimized "click‐like" chemistry and therefore are REadily AvailabLe (REAL),^10^ that is, can be synthesized fast (3–4 weeks) and with 80% success rate, making them almost as accessible as on‐shelf compounds. (3) *More accurate and reliable structure‐based virtual screening methods* have been developed that routinely yield 10%–40%, and sometimes even higher hit rates,^11^ especially when applied to ultra‐large libraries.

Screening of 10 billion compounds at supercomputer centres or computing clouds, however,even at the rock bottom computing rates of ¢1/CPU hour, can easily run into a million‐dollar bill, while expansion to Terra‐scale libraries would be out of reach entirely. Several approaches have been suggested to address the scalability of screening of ultra‐large REAL space libraries, which became a key bottleneck for virtual screening. Some methods, like VirtualFlow, perform iterations from fast and crude to more slow and elaborate docking^9^, ^12^; others, like MolPAL, iterate docking and machine learning (ML) steps.^13^ These methods do reduce calculation costs several‐fold, enabling screens of up to 1.4B molecules; however, their linear scaling with the number of compounds would make them impractical for 10B or more compounds.

A new solution recently developed in our lab^14^ helps to resolve this scaling bottleneck by taking advantage of the modular nature of REAL Space libraries (Figure 1). The method, called Virtual SYNThone Hierarchical Enumeration Screening, or V‐SYNTHES, first docks and screens a library of chemical fragments, representing all building blocks (synthons) and chemical reaction scaffolds of REAL. Wherethe reaction scaffold can combine various synthons in two or three positions, only one position is filled with a synthon, while others are "capped" by a methyl or phenyl group. This initial fragment library is small, just about 1‐2 million compounds, but it represents the full diversity of the 10–20 billion REAL Space. The top‐scoring 0.1% of fragments that are predicted to bind well into the target pocket are then iteratively appended with all possible second (and third synthons if appropriate), and these intermediate libraries are screened at each iteration against the target pocket. At the final step, full molecules from REAL Space are docked, and the best ∼100 candidates are selected for synthesis and testing. Note that capping of the scaffolds with dummy atoms is critical for accurate predictions because reactive groups of building blocks and scaffolds often create strong fake interactions that are not present in the corresponding full molecule. Another important part of the algorithm is the assessment of the fragment binding pose in the target, which prioritizes hits that have their minimal caps pointed into a region of the pocket where the fragment has space to grow.

The V‐SYNTHES technology was applied in this study to discover new chemotypes for cannabinoid receptors (CBRs) antagonists and ROCK1 kinase inhibitors.^14^ For cannabinoid antagonists, chemical synthesis and experimental testing of novel compounds predicted by V‐SYNTHES resulted in hit rate as high as 33%, including 14 submicromolar ligands. This dramatically improved over a standard virtual screening of the Enamine REAL diversity subset, which required approximately 100 times more computational resources. Moreover, selected analogues of the best hits further improved potencies and affinities reaching sub‐nanomolar range and ∼100‐fold CB2/CB1 selectivity. A similar hit rate was obtained for the ROCK1 kinase, where V‐SYNTHES approach needed only 21 compounds synthesized and tested to detect a hit in the nanomolar range, supporting the ability of the approach to rapidly identify novel lead compounds.

Scaling up with future libraries, V‐SYNTHES approach can easily accommodate the rapid growth of the Enamine REAL Space and similar combinatorial libraries, for example, developed by WuXi's GalaXi Space, Otava's CHEMriya, and in‐house virtual libraries developed by pharmaceutical companies. The advantage of the modular V‐SYNTHES approach is that the computational cost of screening grows linearly with the number of synthons, which is much smaller than the number of full ligands in the library. This scaling advantage is especially strong for 3 and higher component reactions. For Enamine REAL alone, expansion to 4‐5 component reactions space, as well as tangible building blocks (DREAM), should already result in Terra‐scale chemical space (10^12^ –10^15^ compounds), bringing it closer to the estimated 10^20^–10^24^ size of the drug‐like chemical space.^15^ The larger the explored chemical space, the more hits and the higher quality hits could be expected. Moreover, by selecting thousands of diverse virtual hit candidates, computational chemists could further filter the list by predicted physicochemical and pharmacodynamic properties (e.g., oral permeability), and still get thousands of high affinity hits, but with improved drug‐like properties, suitable for in vivo probes and drug candidates.

**FIGURE 1:**
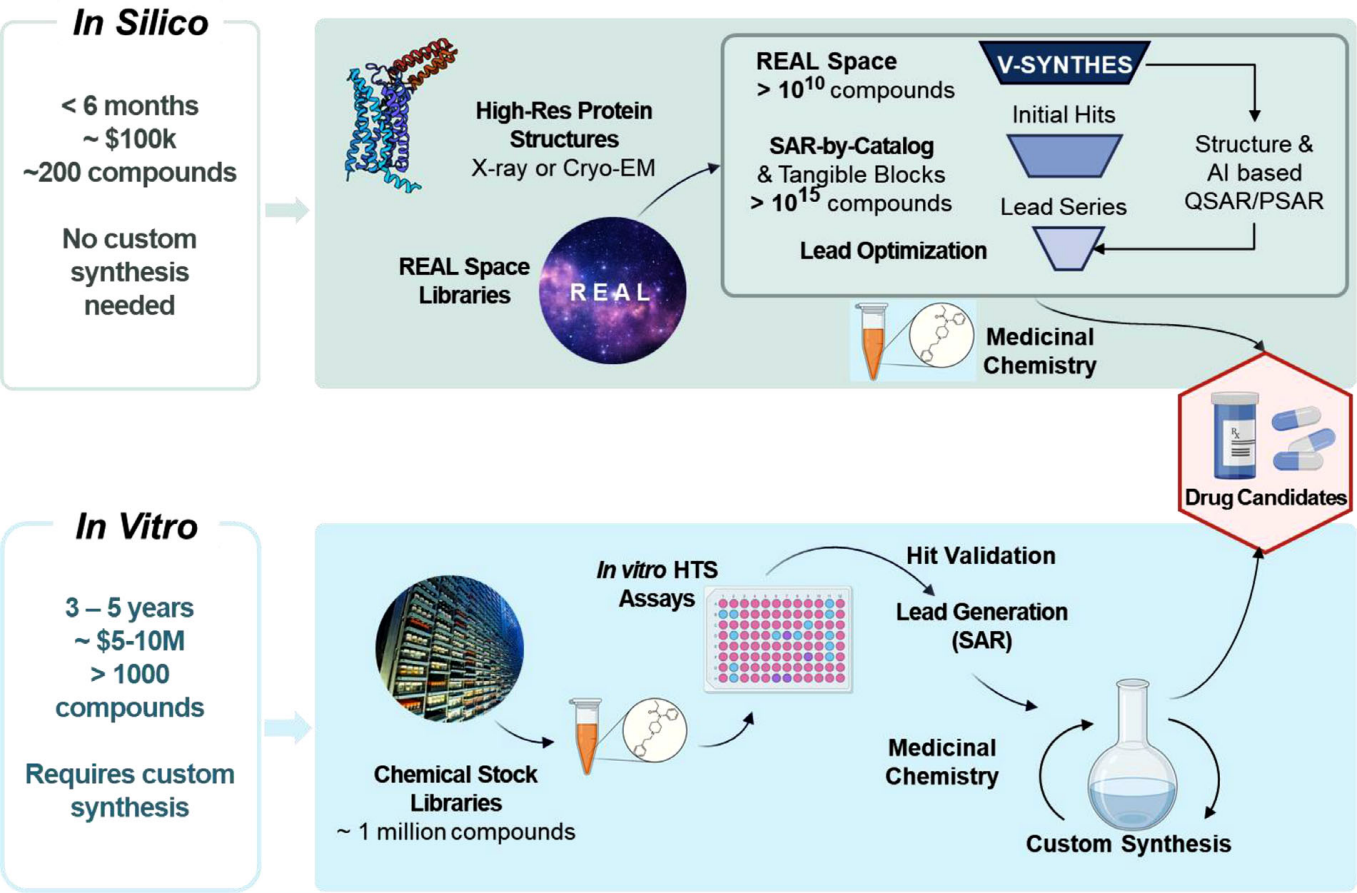
Flowchart of in silico drug discovery with V‐SYNTHES, as compared to traditional HTS‐driven approach. Figure created using biorender.com

Most importantly, approaches like V‐SYNTHES are easily adaptable to any targets with known protein structure and are currently being validated for a variety of GPCRs, kinases, and other target classes. Because the structure‐based methods (unlike machine learning) do not rely on known ligands to build a predictive model, they should apply to many less studied targets, for example, orphan receptors, facilitating discovery of selective in vivo pharmacological probes for new target validation. This is critical for illuminating druggable genome,^16^ rapid development of treatments for emerging and rare diseases,^12^ and adjusting small‐molecule drug discovery for personalized medicine.^17^

## CONFLICT OF INTEREST

V.K. is part of a provisional patent on V‐SYNTHES method (application no. 63159888, University of Southern California).
